# Notes on the Newer Remedies, Their Therapeutic Applications and Modes of Administration

**Published:** 1893-04

**Authors:** 


Notes on the Newer Remedies, Their Therapeutic Applications and Modes of Administration. By David Cerna, M. D., Ph. D. Philadelphia: W. B. Saunders, 913 Walnut Street, 1893. Cloth. Price, $1.25.

So many new remedies have been added to the materia medica of the old school of medicine within the last few years, especially among the coal-tar derivatives, that a book of the character denoted by the title of the volume now under notice, seems to have been imperatively demanded. The present work seems to fulfill the demand, and must prove highly useful to practitioners of the regular school.

It is even useful to homœopathists, who, as well-informed physicians, wish to know the essential nature and origin of such bodies as phenacetine, antipyrine, and other drugs constantly spoken of by adherents of the other school of medicine. It is a small book of one hundred and seventy-five pages.

